# Hernia—Ferguson’s Operation

**Published:** 1899-08

**Authors:** L. W. Hollis

**Affiliations:** Abilene, Texas


For the Texas Medical Journal.

### Hernia—Ferguson's Operation.

BY L. W. HOLLIS, M. D., ABILENE, TEXAS.

Seeing in your last issue a new operation for hernia, by Dr. Lanphear, of St. Louis, prompts me to write you on this subject, thinking, perhaps, it would be of interest to the medical and surgical profession throughout the State.

Dr. Lanphear's operation, like that of Bessini, Halstead, Czerny, Macewan and others, has its short comings and faulty features. I wish to submit to you an ideal operation for the radical cure of oblique inguinal hernia as done by Dr. Alexander Hugh Ferguson, of Chicago. It has been my pleasure to be with Dr. Ferguson during the months of May and June, and I saw him do his typical operation three times. It impressed me very favorably; in fact, impressed me as being *the* operation for the radical cure of hernia.

A typical operation for the radical cure of hernia, as done by Dr. Ferguson, is one that places all the structures involved in the same relationship to one another as they are present in a normal person. The operations, as we are all aware, that have hitherto been produced to cure hernia fall far short of being typical. A careful analysis of failures, a painstaking research for hidden truths and a discernment of contestable premises are ever before the surgeon who hopes for more success, new discoveries and lasting operative procedure.

I will now give the operation as I understand it, step by step, and leave you to judge as to its merit. He first began by making a semi-lunar skin incision, beginning the incision over Poupart's ligament one and one-half inches below the anterior superior spinous process of illium; extending inward and downward in a semi-lunar manner, circumventing the internal abdominal ring, and terminating it over the conjoined tendon near the pubic bone, cutting carefully backwards with a sharp knife and exposing the vessels and picking them up with forceps before severing them, thus preventing blood staining of the tissues. Having passed through the skin, two layers of superficial fassia, fat between them and superficial epigastric vessels down to the aponeurosis of the external oblique muscles, he takes a pledget of gauze and with it turns down the skin flap, subjacent fat and fascia, over the thigh. This proceeding, as you well know, brings into view the aponeurosis of the external oblique muscle, the external abdominal ring with its pillars and intercolumnar fascia, the hernial sac (if it has descended through the internal ring), external surface of Poupart's ligament and superficial vessels.

Now, cut through the external abdominal ring and intercolumnar fascia, separating the longitudinal fibres of the aponeurosis of the external oblique muscle directly over the inguinal canal, far beyond the internal ring, over the surface of the internal abdominal oblique muscle, and up under the skin to a point nearly opposite the anterior superior spine of illium. The delicate fibres are now encountered and severed. Retract the aponeurosis of the external oblique muscle and thereby bring into sight the deep structures, viz.: the contents of the inguinal canal, the whole sac with its adhesions, the spermatic cord, illio-inguinal nerve, internal abdominal ring (usually enlarged), frequently an accumulation of subserous fat, the cremasteric muscle, conjoined tendon, internal oblique muscle and its deficient origin at Poupart's ligament, transversalis fascia and the internal surface of Poupart's ligament. Dr. Ferguson claims "that the congenital deficient origin of the internal oblique and transversalis muscles is one of the most frequent and important causes of oblique inguinal hernia."

He now dissects the sac carefully from the cord and internal ring, opening the sac. (He opens the sac always to inspect contents, ligates it with cat gut, cuts off and lets stump drop into cavity.) If the sac be congenital he divides it in two, the distal half to form the tunic for the testicle, and the proximal to be treated as mentioned above. If omentum is found in sac, it is liberally withdrawn, tied en masse, cut off and covers stump with its own peritoneum, and returned within the abdomen. This he claims decreases the intra-abdominal pressure and lessens the tendency to hernia. When he opens the sac he always puts the patient in Trendelenburg position to prevent protrusion and injury to the intestines.

He does not disturb the cord—lets it alone; claims that the testicle will come to grief by the unnecessary transplanting of the cord. He says, "tearing the cord out of its bed is without one anatomic reason to recommend it, a physiological act to suggest it, an etiological factor in hernia, congenital or acquired, to indicate it, nor brilliant surgical result to justify its continuance." If an abnormal amount of subserous adipose tissue is present he cuts it away.

He now restores the structures to their normal positions. The transversalis fascia forms the internal ring. As you know, in hernias its fibres have become more or less stretched above and around the cord. The ring in consequence is abnormally large and the fascia bulges outwards. To rectify this condition, he takes up the slack in the fascia and makes an accurately fitting ring for the cord, by means of a suture of cat gut, interrupted or continuous, being careful not to injure the deep epigastric vessels or pass the needle too deeply in the direction of the large illiac vessels.

He now catches the internal abdominal oblique and transversalis muscles, suturing them to the internal aspect of Poupart's ligament, restoring them to their normal origin. He freshens the lower border of muscles and surface of Poupart's ligament to insure firm union—suturing fully two-thirds down Poupart's ligament (which is the normal origin of this muscle in the female). Takes care not to split Poupart's ligament by grasping with the needle the same longitudinal fibres each time. It is surprising how easily these two structures come together without the least discernible tension, and it is very gratifying to observe how perfectly these powerful muscles cover and protect the internal abdominal ring and inguinal canal.

He now brings together the separated edges of the aponeurosis of the external oblique muscle, restoring the external abdominal ring. Now closes skin wound with silk worm gut and horse hair sutures, completing a beautiful and simple operation.

The commendable features of it are:

1. The different structures in the abdominal wall are placed in their normal relationship, the tying of the sac restores the normal rotundity of the peritoneum.

2. The suturing of the transversalis fascia forming a new internal ring at the same time obliterates the hernial infundabuliform process.

3. Sewing the internal oblique and transversalis muscles to Poupart's ligament secures a normal origin for them, and they then form perfect protection to the internal ring, cord and canal.

4. The suturing of the separated fibers of the aponeurosis of the external oblique protects the underlying muscles and cord, while the skin flap covers all.

I call your attention to the fact that the four sutures are not opposite each other, thus securing an overlapping of the weak parts. The semi-lunar incision has of itself great advantages. The hernial area is uncovered as in no other way, thus offering an accurate observation of the structural relationship, etiological factors and pathological conditions. There is less danger of skin infection extending to the deep structures. Should, unhappily, a return of the rupture occur, there is no scar over it, and a truss can be better worn. Of all the operations that I have done or seen done this is the simplest and easiest to execute, and there is good scientific reason furnished for every step in the operation. Dr. Ferguson tells me that he has done this operation sixty-four times, and sixty-one out of the sixty-four united by primary union.

Dr. Ferguson is a thorough gentleman, and a surgeon of the first rank, and while he has only been in Chicago four or five years, he today ranks with Senn, Murphy, Ries and Martin as an operator. I had the pleasure of seeing him do many fine operations, such as hystorectomies, both vaginal and abdominal.

